# Genome mining reveals unlocked bioactive potential of marine Gram-negative bacteria

**DOI:** 10.1186/s12864-015-1365-z

**Published:** 2015-03-07

**Authors:** Henrique Machado, Eva C Sonnenschein, Jette Melchiorsen, Lone Gram

**Affiliations:** Novo Nordisk Foundation Center for Biosustainability, Technical University of Denmark, Kogle Allè 6, DK-2970 Hørsholm, Denmark; Department of Systems Biology, Technical University of Denmark, Matematiktorvet bldg 301, DK-2800 Kgs Lyngby, Denmark

**Keywords:** AntiSMASH, Genome mining, *Pseudoalteromonas*, Secondary metabolites, Vibrionaceae

## Abstract

**Background:**

Antibiotic resistance in bacteria spreads quickly, overtaking the pace at which new compounds are discovered and this emphasizes the immediate need to discover new compounds for control of infectious diseases. Terrestrial bacteria have for decades been investigated as a source of bioactive compounds leading to successful applications in pharmaceutical and biotech industries. Marine bacteria have so far not been exploited to the same extent; however, they are believed to harbor a multitude of novel bioactive chemistry. To explore this potential, genomes of 21 marine Alpha- and Gammaproteobacteria collected during the Galathea 3 expedition were sequenced and mined for natural product encoding gene clusters.

**Results:**

Independently of genome size, bacteria of all tested genera carried a large number of clusters encoding different potential bioactivities, especially within the Vibrionaceae and Pseudoalteromonadaceae families. A very high potential was identified in pigmented pseudoalteromonads with up to 20 clusters in a single strain, mostly NRPSs and NRPS-PKS hybrids. Furthermore, regulatory elements in bioactivity-related pathways including chitin metabolism, quorum sensing and iron scavenging systems were investigated both *in silico* and *in vitro*. Genes with siderophore function were identified in 50% of the strains, however, all but one harboured the ferric-uptake-regulator gene. Genes encoding the syntethase of acylated homoserine lactones were found in Roseobacter-clade bacteria, but not in the Vibrionaceae strains and only in one Pseudoalteromonas strains. The understanding and manipulation of these elements can help in the discovery and production of new compounds never identified under regular laboratory cultivation conditions. High chitinolytic potential was demonstrated and verified for *Vibrio* and *Pseudoalteromonas* species that commonly live in close association with eukaryotic organisms in the environment. Chitin regulation by the ChiS histidine-kinase seems to be a general trait of the Vibrionaceae family, however it is absent in the Pseudomonadaceae. Hence, the degree to which chitin influences secondary metabolism in marine bacteria is not known.

**Conclusions:**

Utilizing the rapidly developing sequencing technologies and software tools in combination with phenotypic *in vitro* assays, we demonstrated the high bioactive potential of marine bacteria in an efficient, straightforward manner – an approach that will facilitate natural product discovery in the future.

## Background

The discovery and development of new molecules for medical treatment is in great need as the 21^st^ century unfolds. Drug-resistant pathogenic microorganisms are becoming a significant threat to public health and the pharmaceutical discovery pipelines have not been delivering the amount of new drugs required for efficient disease treatment [1-3]. Chemical synthesis has developed to be faster and cheaper as compared to biological screenings of organisms and extracts, however, chemical synthetic libraries have not provided the expected novel drugs and a high percentage of new chemicals that are introduced into the markets by pharmaceutical companies are actually derived from natural products [4]. Most of the natural products identified are produced by non-ribosomal peptide synthases (NRPSs) and/or polyketide synthases (PKSs) [5,6]. NRPSs and PKSs are multifunctional modular enzymes that assemble small molecules from monomers like pearls on a string. Both enzyme types have core domains responsible for the recognition of the monomer, attachment to the enzyme, condensation and chain-termination. Additionally, domains for tailoring the monomers can be present. In case of PKSs, such as in fatty acid synthesis, the monomers are acyl-CoAs, while NRPSs connect naturally occurring as well as unnatural amino acids to peptide chains. This wide range of possible subunits and the possibilities of their combinations lead to the great diversity of polyketides (PKs) and non-ribosomal peptides (NRPs) [7].

For the last century, soil microorganisms have been isolated and screened intensively to discover novel antibiotics and other drugs, and, in total, microorganisms have supplied more than 80.000 natural products [8]. Today, terrestrial *Streptomyces* is probably the best exploited genus with respect to secondary metabolites [9-13]. *Streptomyces* species produce a great diversity of compounds with antifungal (nystatin, natamycin, amphotericin), antibacterial (chloramphenicol, streptomycin, holomycin) and antiparasitic (ivermectin) activity [14]. Also new cultivation approaches are being used to culture new taxa, which potentially can be a source of novel compounds, as the recently described case of teixobactin [15].

Even though scientists have started to explore several other habitats than the terrestrial, the marine environment stands out as a hitherto under-explored niche for new bioactive molecules [6,16-19]. Previous studies have indicated that since the environmental conditions are very different from terrestrial habitats, novel compounds and chemical classes are present, and indeed some marine natural products are characterized by the unique marine factors such as halogenation [20-22]. Marine natural products have been isolated and identified from several different sources such as algae, sponges or molluscs, however, several recent studies have attributed the production of many of these compounds to microorganisms associated with the eukaryotic producer previously identified [23], bringing marine microorganisms to the spotlight of natural product discovery.

Following the success of terrestrial streptomycetes as producers of natural products, several researchers have focused their search on marine actinobacteria and the discovery of the first truly marine actinobacterium *Salinispora* has provided a number of very interesting bioactive compounds, including the anti-cancer compound salinosporamide [24,25]. Also, subsequent mining of the genome demonstrated an impressive number of potentially bioactive gene clusters [16]. The Gram-negative proteobacteria have generally been thought to have less potential for the production of bioactives than actinobacteria, however, several bioactive compounds have been isolated from the marine genus *Pseudoalteromonas* and more recently also from strains of the *Roseobacter* clade and the Vibrionaceae family [19,26-29].

Hitherto, the vast majority of bioactive compounds have been found using a classical bioassay-guided process, however, this bioprospecting of drugs is expensive and time-consuming, and re-discovery of known compounds is, despite dereplication steps, a major challenge. The process of drug discovery is currently undergoing changes as a result of the rapid developments in sequencing technology and synthetic biology. The number of whole microbial genomes and metagenomic data made publicly available is increasing exponentially and therefore, (meta)genome mining has become an extremely attractive tool for drug discovery [2,3,16,30,31]. It has led to the development of new bioinformatic tools used for screening and identification of the genetic background of the bioactivities including gene clusters responsible for the production of the novel molecules. Many of these clusters are probably silent under most laboratory culture conditions and require induction [32]. Several of the bioinformatic tools have been designed to search specifically for PKS and NRPS clusters, of which the structure is conserved. Several recent reviews provide a comparison between different tools, considering their *modus operandi* [30,31].

AntiSMASH version 2 is a strong comprehensive tool [30] and includes the use of several of the other tools available, such as the CLUSEAN tools [33], NRPSpredictor1/2 [34,35] and a method by Minowa et al. [36]. Even though the occurrence of misidentifications is quite common, it is preferable to “over-identify” rather than missing potential gene clusters [30]. Therefore, complementing antiSMASH analysis with more specific tools aids in the gene cluster identification. In this study, we used three other tools: BAGEL3 for the identification of bacteriocins [37]; NapDos for the identification of keto-synthase (KS-domains) and condensation domains (C-domains) [38]; and NP.search for the identification of whole gene clusters that may be composed of several KS- and/or C-domains [39]. C- and KS-domains catalyze the chain formation of the subunits (peptides or acyl-CoAs), respectively and a high number of these domains reflects the richness of bonds possibly made by an organism and the degree of diversity on non-ribosomal peptide synthesis.

The strains investigated in this genome mining study were isolated during the Galathea 3 global expedition in 2006/7. Antagonistic activity towards the human pathogen *Staphylococcus aureus* and the fish pathogen *Vibrio anguillarum* were the main selection criteria [19]. The Galathea 3 bacterial collection has been used in previous studies where identification of new bioactive compounds has been successful. For instance, *Photobacterium halotolerans* strain S2753 produces novel compound families, the solonamides and ngercheumicins, which interfere with virulence regulation in *S. aureus* [40-42]. *Vibrio nigripulchritudo* strain S2604 produces a novel siderophore: nigribactin [43]. However, also several known antibiotic compounds were re-discovered, for instance, S2753 produces holomycin [28], an antibiotic previously only isolated from terrestrial streptomycetes, and *Vibrio coralliilyticus* S2052 produces andrimid [28]. Also, in pigmented *Pseudoalteromonas*, we have re-identified a range of antibiotic compounds (indolmycin, pentabromopseudilin, prodigiosin) [44,45].

During the last five years, we have demonstrated that marine Gram-negative bacteria produce an array of antibiotic and anti-virulence compounds [19,28,29,40,41,43-45] and here, we ask the question if the classical bioprospecting approach had fully revealed the potential of these bacteria. We present an *in silico* study of different marine bacterial genomes, which were analyzed using several of the prediction tools developed for the identification of secondary metabolism pathways, namely antiSMASH, NapDos, Np.search, and BAGEL3 [37,46,47]. We combined the genome mining with phenotypic evaluation of molecules potentially involved in production or regulation of bioactive compounds; namely, quorum sensing signals, siderophores and chitinases.

## Results and discussion

### Marine bacterial genomes – genome size

The genomes were assembled using CLC Genomics Workbench 7 (CLC bio, Aarhus, Denmark) to obtain contig-based draft genomes of the strains. These draft genomes were then annotated using the Rapid Annotation using Subsystem Technology (RAST) [48,49]. The subsequent analysis of the genomes was performed using CLC Main Workbench 7 (CLC bio, Aarhus, Denmark).

The genome size varied between 3.6 and 6.2 Mb in the 21 sequenced strains (Table 1). In the six Vibrionaceae, the genomes varied between 4 and 6.2 Mb, and the genomes of the eight *Pseudoalteromonas* spp. ranged from 4.1 to 6.1 Mb. The genomes of the five strains from the Rhodobactereaceae family were slightly smaller; from 3.6 to 4.8 Mb. The *in vitro* bioactivity (antibacterial activity measured as zone size) [19] did not correlate to the genome size (Table 1).

**Table 1:** **Potential for production of bioactive secondary metabolites from 21 marine bacterial strains**

**Strain**	**Species**	**Genome size (Mb)**	**Antibacterial activity**	**AntiSMASH (total hits)**	**BAGEL3**	**NapDos**		**NP.search**			
						**KS-domains**	**C-domains**	**NRPS**	**PKS**	**Mix**	**Trans PKS**
**S2753**	*Photobacterium halotolerans*	5.5	yes	12	0	3	19	1	0	1	0
**S2052**	*Vibrio coralliilyticus*	5.4	yes	7	2	7	13	2	0	2	0
**S2043**	*Vibrio coralliilyticus.*	5.4	yes	7	2	7	13	2	0	2	0
**S2604**	*Vibrio nigripulchritudo*	6.2	yes	9	0	6	17	1	0	0	0
**S2394**	*Vibrio neptunius*	5.2	yes	6	1	4	12	1	0	1	0
**S2757**	*Vibrio* sp*.*	4.0	no	2	0	5	0	0	0	0	0
**S2040**	*Pseudoalteromonas piscicida*	5.3	yes	14	1	8	58	7	0	1	0
**S2724**	*Pseudoalteromonas piscicida*	5.2	yes	10	1	7	30	2	0	2	0
**S816**	*Pseudoalteromonas agarivorans*	4.4	no	2	0	5	0	0	0	0	0
**S3258**	*Pseudoalteromonas ruthenica*	4.1	yes	3	0	5	0	0	0	0	0
**S3137**	*Pseudoalteromonas ruthenica*	4.1	yes	3	0	5	0	0	0	0	0
**S4054**	*Pseudoaltermonas luteoviolacea*	6.1	yes	20	1	14	48	3	0	4	1
**S2471**	*Pseudoalteromonas rubra*	5.8	yes	17	2	12	56	3	0	2	1
**S2151**	*Halomonas* sp*.*	5.2	no	5	0	7	0	0	0	0	0
**S3726**	*Marinomonas* sp.	5.4	yes	5	0	6	17	2	0	0	0
**S2292**	*Spongiobacter* sp.	4.7	yes	5	1	3	3	0	0	0	0
**S4079**	*Loktanella* sp.	3.6	no	5	1	3	3	0	0	0	0
**S4493**	*Paracoccus* sp.	4.0	yes	11	1	3	2	0	0	0	0
**S1942**	*Ruegeria mobilis*	4.8	yes	8	1	4	1	0	0	0	0
**F1926**	*Ruegeria mobilis*	4.6	yes	9	0	5	1	0	0	0	0
**DSM17395**	*Phaeobacter inhibens*	3.8	yes	9 + 1	0	4	1	1	0	0	0

It has been suggested that the potential for production of secondary metabolites would be related to genome size [11,50,51], with a larger genome allowing more genes to be allocated to secondary metabolism. This notion was to some extend developed by studies of the genus *Streptomyces* which is a prolific producer of secondary metabolites and has relatively large genomes of approx. 8 Mb in size as compared to other bacteria. This understanding is changing, as the marine actinomycete *Salinispora* sp. has a genome size of approx. 5 Mb, of which approx. 10% is dedicated to secondary metabolism, whereas only approx. 8% of the genome of *Streptomyces coelicolor* has been reported as dedicated to secondary metabolism [11,16].

### Identification of gene clusters potentially encoding secondary metabolites

The genomes were mined using bioinformatic tools for the identification of clusters involved in secondary metabolism, namely antiSMASH, NapDos, Np.search, and BAGEL3 [37-39,47]. We found a high genetic potential for secondary metabolite production also in Gram-negative marine bacteria with genome sizes ranging from 4 to 6 Mb, with some strains reaching the considerable number of eight distinct PKS/NRPS clusters (Table 1 – NP.search). However, some strains with similar genome size harbored none or very few potential bioactive clusters and thus, there was no clear correlation between genome size and number of secondary metabolism gene clusters. Some strains, such as *V. nigripulchritudo* S2604 or *Halomonas* sp. S2151, with larger genomes had a low number of hits; and also contrarily, strains with smaller genomes had a greater number of hits e.g. *P. piscicida* strains S2040 and S2724 (Table 1).

#### Bioactivity potential - NRPS/PKS

The presence of gene clusters likely encoding bioactive compounds is spread among the different families of Alpha- and Gammaproteobacteria. Although our collection is limited in number, it appears that the Gammaproteobacteria class is richer in NRPS and PKS clusters than the Alphaproteobacteria. The analysis using NapDos and NP.search, in general, identified the same number of potential bioactive gene clusters. A higher frequency of KS- and C-domains was identified in pigmented *Pseudoalteromonas* strains (S2040; S2724; S4054; S2471) followed by Vibrionaceae, with the exception of S2757 (no hits), and *Marinomonas* sp. S3726 (high number of hits).

Some species in the Rhodobactereaceae family (*Ruegeria mobilis* and *Phaeobacter inhibens*) are capable of inhibiting a wide range of other bacteria [52-54]; however, in general, few secondary metabolites have been identified in these strains [54-57]. Here, we show that using bioinformatics tools a few clusters could be identified, but still the bioactive potential harbored in the genome of these genera appears much lower than that observed in Gammaproteobacteria.

A number of strains that were not antagonistic in agar-based assays were included in the analysis and these contained only few gene clusters potentially coding for secondary metabolites (Table 1). This was the case for *Vibrio* sp. S2757 and *P. agarivorans* S816, for which antiSMASH identified only two potential clusters (Table 1).

Another interesting group of strains included those that received five hits in total in the antiSMASH analysis. This includes the bioactives *Marinomonas* sp*.* S3726 and *Spongiobacter* sp. S2292 and the non-bioactives *Halomonas* sp. S2151 and *Loktanella* sp. S4079. Although, all of them had a considerably lower number of hits in the antiSMASH analysis than the pigmented pseudoalteromonads and the vibrios, the results of the other mining tools (NaPDoS) demonstrate that *Marinomonas* sp. S3726 has a great potential with 6 KS- and 17 C-domains identified (Table 1). Thus, the sole number obtained by one given analysis tool may not reflect the whole potential of the organism, and complementary analysis should be performed to ensure discovery of the full bioactive potential. This should also be done to avoid further work on clusters that may not be true secondary metabolite clusters, it appears from the analysis that NapDos and NP.search tools seemed to identify only a subset of the NRPS/PKS clusters identified by antiSMASH.

**Figure 1:**
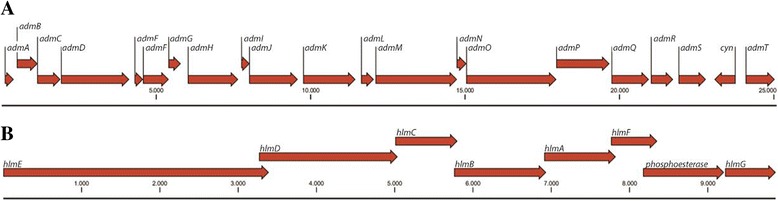
**Previously known clusters identified in the studied marine bacteria, using genome mining.** Andrimid gene cluster from *V. coralliilyticus* S2052 **(A)**; Holomycin gene cluster from *P. halotolerans* S2753 **(B)**.

The potential for secondary metabolite production in the strains is clearly much larger than so far identified by bioassay-guided fractionation. For instance, the PK/NRP hybrid andrimid has been identified as the bioactive compound in *V. coralliilyticus* S2052 [28,29,58,59]. The genome mining identified the gene cluster likely encoding for andrimid production genes (Figure 1(A)). Also, we found at least three more NRPS clusters using antiSMASH, NapDos and NP.search (Table 1). Similarly, in *P. halotolerans* S2753, the dithiolopyrrolone holomycin was identified in extracts [28] and the corresponding gene cluster was found by the bioinformatic tools used (Figure 1(B)); again, four more NRPS/PKS clusters were found using antiSMASH, although only one more was discovered when using NP.search (Table 1). As indicated, we and others have identified several bioactive compounds from pigmented pseudoalteromonads and here we also identified the respective gene clusters for indolmycin [44], violacein [60] and pentabromopseudilin [5,61,62]. However, the pigmented pseudoalteromonads contained a large number of potential bioactive clusters, including a very high number of C-domains as compared to the other studied strains (NapDos – Table 1).

#### The case of Pseudoalteromonas ruthenica

In *in vitro* assays, *Pseudoalteromonas ruthenica* is highly antagonistic against *S. aureus* and *V. anguillarum* causing large clearing zones in agar-based screening assays [19]. However, we have not been able to identify the compound(s) responsible for this inhibition by bioassay-guided fractionation and anticipated that genome mining would reveal potential bioactive gene clusters. AntiSMASH identified three gene clusters (one for siderophore and two for bacteriocin biosynthesis), but only the siderophore cluster was correctly identified, whereas the bacteriocin-related clusters were misidentified and encoded the flagella operon and a cluster encoding for hypothetical proteins, a muramoyltetrapeptide carboxypeptidase and a 2,3,4,5-tetrahydropyridine-2,6-dicarboxylate N-succinyltransferase, involved in the biosynthesis of peptidoglycan and lysine, respectively.

A second analysis of the *P. ruthenica* strains with antiSMASH based on PFAM domain probabilities increased the number of potential gene clusters from three to thirteen. Mainly clusters encoding for acyl carrier proteins were identified, but we also identified some biosynthetic clusters such as lipopolysaccharide, capsular polysaccharide, legionaminic acid and fatty acid biosynthesis. From all the clusters, only one matched with the RAST annotation as behaving an open reading frame (ORF) encoding a non-ribosomal peptide synthase. Yet this ORF was only 663 bp, and when we blasted the predicted aminoacid sequence against the NCBI protein database, it presented a high similarity with a methionyl-tRNA formyltransferase and not to an NRPS. In agreement with these were NapDos and NP.search, which did not identify any potential bioactive clusters (Table 1). This reduces the likelihood that the clusters identified by antiSMASH using PFAM domains are actually clusters responsible for the production of bioactives.

In genome mining, the identification of clusters likely involved in secondary metabolism, such as NRPS and PKS, have been used as a measure of the potential for finding novel natural bioactive compounds, including antibiotics [63]. Yet, all the bioinformatic tools used to search for the biosynthetic capabilities and potential of *P. ruthenica* failed. This might be the case because the antagonistic activity is due to other biosynthetic pathways as is for instance the case with the antibiotic tropodithietic acid produced by some *Roseobacter* clade bacteria [64]. Also, it can be attributed to limitations in the prediction algorithms. The prediction algorithms of the bioinformatic tools are to some extend based on identification of known biosynthetic activities and one could speculate that truly novel biosynthetic pathways would not be identified. To identify the core genes of a biosynthetic pathway, most of the tools available use profile-HMMs or alignments of conserved domains in biosynthetic enzymes [30]. This is a problem in the identification of non-standard pathways and antiSMASH has therefore implemented an algorithm to identify the distribution of protein domains usually associated with secondary metabolites [30], increasing the probability of identification of clusters responsible for secondary metabolites production. This not only increases the number of hits, but also the time needed for evaluation of the clusters, raising the question of the feasibility of using genome mining in groundbreaking discoveries.

#### Bacteriocins

The number of clusters identified by antiSMASH as bacteriocins varied between one and five in each strain, with an average of two clusters per strain. However, when the genomes were analyzed using the prediction tool BAGEL3 [37], the presence of bacteriocin-related genes was only confirmed in a few strains. The distribution of bacteriocin clusters did not follow a particular pattern with respect to genera or species.

It seems evident that the specific prediction tools are more accurate in identifying their defined target; therefore, BAGEL 3 being most probably a better indicator of the number of bacteriocin-related genes than antiSMASH itself. This becomes more evident from the *P. ruthenica* case, where random genes were classified as bacteriocins (see above).

### Acyl homoserine lactones

Four of the 21 strains induced a clear response in the AHL (acyl homoserine lactone) biomonitors (Table 2). Three strains, *Vibrio* sp. S2757, *Paracoccus* sp. S4493 and *P. luteoviolaceae* 4054 induced both monitors whereas *P. inhibens* DSM17395 induced only *A. tumefaciens*. This is in agreement with previous studies where also *Phaeobacter* sp. strain S27-4 induced *A. tumefaciens* and chemical analysis identified 3-hydroxy-decanoyl-homoserine lactone [64]. Interestingly, antiSMASH detected homoserine lactone synthases in three of these four strains but not in *Vibrio* S2757. The response in the monitor strains could be caused by other compounds, such as diketopiperazines that have been demonstrated to induce the AHL monitors [65]. The same could be true for the extracts of the *V. coralliilyticus* strains S2052 and S2043, which resulted in a weak reaction in *C. violaceum*, and the genomes did not contain an AHL synthase gene. AntiSMASH detected AHL synthase genes in three strains (*Loktanella* sp. and two *Ruegeria mobilis*) where no AHLs were detected by the monitors (Table 2). These genes could potentially encode novel AHLs not being in the detection range of the used biological monitors [66]. On the other hand, the bacteria may not have been cultured under conditions allowing the expression of the presumed AHL synthase genes or the AHL concentration produced was below the detection limit. We considered if the potential QS systems could be involved in production of secondary metabolites. In *P. luteoviolaceae*, the AHL synthase gene is adjacent to the gene cluster potentially involved in indolmycin production [67], but in the other five strains the HSL synthase genes detected by antiSMASH were not in proximity to identified natural product gene clusters. However, some were close to genes encoding acyl synthases, alcohol dehydrogenases or proteins containing AMP-binding domains, which may potentially be involved in secondary metabolism. Due to draft genomes with multiple contigs, the association with natural product gene clusters could have been lost in the analyzed sequences.

**Table 2:** **Iron system in the studied strains, comprising** ***in silico*** **and phenotypical results**

**Strain**	**Species**	**Response in AHL monitor**		**AntiSMASH**	**Siderophore (CAS)**	**AntiSMASH**		**Fur**
		**Cv**	**At**	**HSL**		**Siderophore**	**NRPS**	
**S2753**	*Photobacterium halotolerans*	-	-	0	+	1	4	1
**S2052**	*Vibrio coralliilyticus*	(+)	-	0	-	1**	4***	1
**S2043**	*Vibrio coralliilyticus*	(+)	-	0	(+)	1**	4***	1
**S2604**	*Vibrio nigripulchritudo*	-	-	0	-	0	4***	1
**S2394**	*Vibrio neptunius*	-	-	0	(+)	1	3***	1
**S2757**	*Vibrio sp.*	+	+	0	+	1**	0	1
**S2040**	*Pseudoalteromonas piscicida*	-	-	0	+	0	11***	1
**S2724**	*Pseudoalteromonas piscicida*	-	-	0	+	0	5***	1
**S816**	*Pseudoalteromonas agarivorans*	-	-	0	+	1	0	1
**S3258**	*Pseudoalteromonas ruthenica*	-	-	0	+	1	0	1
**S3137**	*Pseudoalteromonas ruthenica*	-	-	0	-	1	0	1
**S4054**	*Pseudoaltermonas luteoviolacea*	+	+	1	(+)	0	11***	1
**S2471**	*Pseudoalteromonas rubra*	-	-	0	(+)	0	9***	1
**S2151**	*Halomonas* sp*.*	-	-	0	(+)	1	0	1
**S3726**	*Marinomonas* sp*.*	-	-	0	+	0	3***	1
**S2292**	*Spongiobacter* sp*.*	-	-	0	-	0	1	1
**S4079**	*Loktanella* sp*.*	-	-	1	(+)	0	1***	1
**S4493**	*Paracoccus* sp*.*	+	+	4	-	0	2	0
**S1942**	*Ruegeria mobilis*	-	-	2	(+)	0	1***	1
**F1926**	*Ruegeria mobilis*	-	-	2	(+)	0	1***	1
**DSM17395**	*Phaeobacter inhibens*	-	+	2	+	1*	1	1

Cv: *Chromobacterium violaceum*, At: *Agrobacterium tumefaciens*, HSL: homoserine lactone, CAS: chrome-azurol-S, + : strong bioactivity, (+) : weak bioactivity, − : no bioactivity detected under the tested conditions, NRPS: including single NRPS clusters and NRPS fusion clusters (e.g. NRPS-bacteriocin, NRPS-ectoine). *Located on a plasmid; **Cluster identified as a siderophore – ectoine cluster; ***At least one NRPS is in proximity to siderophore-associated genes (tonB-dependent receptor etc.).

### Siderophores and iron regulation

Iron is essential for almost all microorganisms being required for key biological processes [68] and is also one of the most important requirements for successful secondary metabolism. The iron levels in seawater are extremely low, and many marine bacteria are able to sequester iron using siderophores that can also serve as a tool in microbial competition. Hence, siderophores are included as secondary metabolites in the antiSMASH search.

To complement the genetic search, we determined siderophore activity using the CAS assay [69]. Pronounced siderophore activity was detected in eight strains and a weak reaction was observed in eight further strains. Only five strains did not show any activity under the tested conditions (Table 2). The NRPS prediction tools, NapDos and NP.search, do not allow detailed prediction of the type of NRPS coding gene, however, antiSMASH is able to distinguish siderophore synthesis genes. The *in silico* analysis using antiSMASH identified putative siderophore gene clusters in five of the eight strains with a clear CAS reaction, and three of the eight with a weak reaction. In one strain, *P. ruthenica*, antiSMASH detected a siderophore synthesis gene, but the CAS assay was negative. In contrast, the CAS reaction was positive for three strains (two *P. piscicida* and one *Marinomonas*) where a siderophore biosynthesis gene was not detected. AntiSMASH predicts siderophore genes using the currently available sequence information on siderophore-producing NRPSs, which are mainly of terrestrial origin. Terrestrial siderophores differ structurally from marine siderophores that are usually associated with fatty acids [68]. We analysed the identified NRPS gene clusters for siderophore-associated genes such as *tonB*-dependent receptor genes. For twelve strains, these siderophore-associated genes were found close to the NRPS gene leading to the hypothesis that this NRPS gene could likely encode a siderophore-producing NRPS. This would demonstrate that all strains based on their genetic information would be capable of scavenging iron using siderophores. To detect this “hidden” activity for the five non-active strains, the strains might require optimization of culture conditions or certain biological cues from the environment. Iron can also be scavenged by other molecules and non-siderophore iron sequestering systems may be operational in the bacteria where siderophore genes were not detected. Indeed, several heme-related proteins were identified among the studied marine bacteria by an annotation-based search (data not shown).

**Figure 2:**
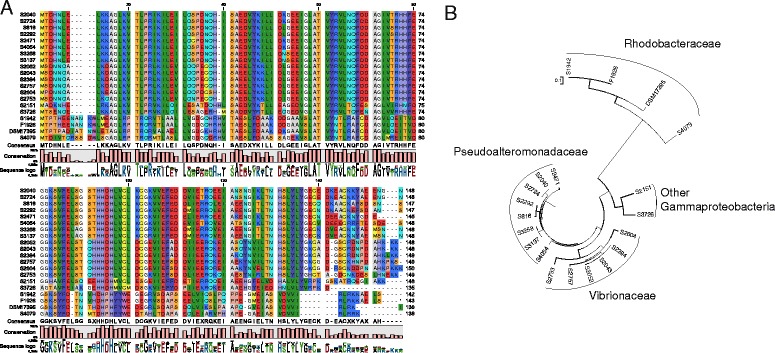
**Analysis of the Fur sequences of the marine bacterial strains used.** Predicted Fur protein sequence alignment **(A)**; Phylogenetic neighbor joining tree using Jukes-Cantor protein distance measurement method **(B)**.

Even though iron is essential for growth, excess of iron can be toxic to bacteria and thus a tight regulation of uptake is crucial for microbial survival [68]. In Gram-negative bacteria, iron regulation is achieved by a repressor protein named Fur (Ferric-iron uptake regulator) which acts at the transcriptional level [70]. A Fur encoding gene could be identified in all the studied strains and the amino acid sequence predicted, with the sole exception of *Paracoccus* sp. S4493 (Table 2; Figure 2(A)). The verified exception of *Paracoccus* sp. S4493 might be due to sequencing limitations, or the fact that this organism has another regulatory protein involved in iron sensing; in fact other uptake regulators for different metals could be identified (e.g. manganese, potassium, zinc, and nickel).

Within the classes of Proteobacteria, the Fur proteins are relatively conserved at the amino acid level, presenting a higher variation at the C-terminus and the N-terminus. Nevertheless, the conserved features such as DNA-binding-α-helix and Fe^2+^ and Zn^2+^ binding domains could be identified [71]. A neighbor joining tree using Jukes-Cantor protein distance measurement method (Figure 2(B)) demonstrates the conservation of closely related species, indicating that the *fur* gene is a phylogenetic trait instead of a random species variation or a product of recent horizontal gene transfer. In fact, the clusters based on protein sequences follow the phylogenetic distribution; the analyzed Alphaproteobacteria sequences form a separate, distant group from the families of Gammaproteobacteria, in which the Pseudoalteromonadaceae and the Vibrionaceae families form two distinct clusters from the other Gammaproteobacteria. The only exception here was the *Spongiobacter* sp. S2292, which clustered together with the *Pseudoalteromonas* spp. This is interesting, since the 16S rRNA sequence (GenBank acc. no. FJ457273.1) would place *Spongiobacter* sp. S2292 closer to the *Endozoicomonas* genus and therefore within the order of Oceanopirillales, in which the species *Halomonas* and *Marinomonas* are also included [72]. This fact brings to question the phylogenetic placement of *Spongiobacter* as it remains an unclassified member of Gammaproteobacteria, and this association indicates a closer association with *Pseudoalteromonas* species than with the other Gammaproteobacteria.

Interestingly, this clustering seems to be specific at species level, even for the *Vibrio* strains studied. Identifying *Vibrio* strains to species level typically requires multilocus sequence analysis [73,74]. We recently showed that the *fur* gene is a good phylogenetic marker (Machado & Gram, submitted) to be added to the multilocus sequencing analysis performed nowadays in e.g. *Vibrio* species definition [73-75] and might also be possibly used in other genera for species differentiation.

### Chitinases and regulation

Chitin is ─ after cellulose ─ the most abundant carbon source on Earth. Enzymes capable of degrading this organic compound are very useful in biotechnological industries. At the same time, chitin is also an important environmental clue influencing regulators of virulence and secondary metabolism [58,76-78]. We have previously shown that an andrimid producing *V. coralliilyticus* S2052 focuses its secondary metabolism exclusively on andrimid when grown on chitin as compared to growth on glucose and casamino acids [58]. This could be coupled with transcriptional changes and we therefore also mined the genomes for chitin catabolic cascade sensor histidine kinase (ChiS) and chitin binding proteins (CBP).

By phenotypic assays, we identified several strongly chitinolytic strains and screened their genomes for chitinase encoding genes. All of the Vibrionaceae and pigmented *Pseudoalteromonas* sp., with the exception of *P. agarivorans* S816, were capable of degrading chitin (Table 3). The genome mining revealed presence of three to nine chitinase encoding genes per strain in the chitinolytic bacteria.

**Table 3:** **Chitinolytic systems in the studied strains, comprising** ***in silico*** **and phenotypic results**

**Strain**	**Species**	**Chitinase activity**	**Chitin**		
			**Chitinase**	**ChiS**	**CBP**
**S2753**	*Photobacterium halotolerans*	+++	3	1	1
**S2052**	*Vibrio coralliilyticus*	++	9	1	2
**S2043**	*Vibrio coralliilyticus*	++	9	1	2
**S2604**	*Vibrio nigripulchritudo*	+	8	2	0
**S2394**	*Vibrio neptunius*	++	7	1	0
**S2757**	*Vibrio sp.*	++	3	1	1
**S2040**	*Pseudoalteromonas piscicida*	++	4	1	0
**S2724**	*Pseudoalteromonas piscicida*	+++	4	0	0
**S816**	*Pseudoalteromonas agarivorans*	-	0	0	0
**S3258**	*Pseudoalteromonas ruthenica*	++	3	0	1
**S3137**	*Pseudoalteromonas ruthenica*	++	3	0	1
**S4054**	*Pseudoaltermonas luteoviolacea*	+	10	0	0
**S2471**	*Pseudoalteromonas rubra*	+	7	0	0
**S2151**	*Halomonas* sp*.*	-	0	0	0
**S3726**	*Marinomonas* sp*.*	-	0	0	0
**S2292**	*Spongiobacter* sp*.*	-	0	0	1
**S4079**	*Loktanella* sp*.*	-	0	0	0
**S4493**	*Paracoccus* sp*.*	-	0	0	0
**S1942**	*Ruegeria mobilis*	-	0	1	0
**F1926**	*Ruegeria mobilis*	-	0	1	0
**DSM17395**	*Phaeobacter inhibens*	-	0	0	0

ChiS: chitin catabolic cascade sensor histidine kinase, CBP: chitin binding proteins. - : no chitinase activity detected, + : low chitinase activity, ++ : medium chitinase activity, +++ : strong chitinase activity.

Chitin-related genes were present in *Vibrio* species, which is likely related to their ecology and close association with crustaceans [79,80]. The pigmented pseudoalteromonads are also often associated with eukaryotic surfaces [44] including organisms containing no chitin. However, several pseudoalteromonads had genes encoding for chitinases and showed prominent chitinolytic activity.

The chitinolytic cascade has previously been studied in *Vibrio* species where its tight regulation was attributed to the hybrid chitin catabolic sensor/kinase (ChiS) together with a periplasmic chitin oligosaccharide binding protein (CBP) [77]. This regulatory system has been shown to regulate expression of 50 genes, most of which involved in chitin catabolism [77]. Furthermore, it has been also shown that natural substrates such as chitin influence secondary metabolite production, such as the induction of their production [58]. We searched for the *chiS* gene, which was present in all of the six Vibrionaceae, one *Pseudoalteromonas*, and two *Ruegeria* (Table 3). The Alphaproteobacteria did not degrade chitin, though two *Ruegeria mobilis* strains harbor the chitin sensor genes.

Interestingly, the ChiS regulator was only present in the *Vibrio* strains, suggesting that transcriptional shaping by chitin could be a trait associated with this family. Changes in secondary metabolism by chitin and the presence of the regulator ChiS requires further studies for confirmation.

## Conclusions

Here, we presented a straightforward, comprehensive genome mining approach analyzing marine bacterial strains for secondary metabolism and associated features such as quorum-sensing, iron acquisition, chitin use as a carbon source and its regulation. The use of complementary tools for genome mining is of great value in narrowing down the potential gene clusters from a large pool obtained by broad prediction software such as antiSMASH. We demonstrated the great potential of marine bacteria for secondary metabolite production, with special focus on *Vibrio* and pigmented *Pseudoalteromonas* species.

## Methods

### Bacterial strains and growth conditions

Bacterial strains used in this study were isolated during the Danish Galathea 3 global research expedition (http://www.galathea3.dk/uk) [19] and selected due to their antagonistic activity against a Gram-negative (*Vibrio anguillarum*) and a Gram-positive (*Staphylococcus aureus*) pathogenic bacterium. Pure cultures of each strain were stored in cryoprotectant solution at −80°C from their isolation until the present study. *Phaeobacter inhibens* DSM17395 was obtained from the German Collection of Microorganisms and Cell Cultures (DSMZ, Germany). Some of the strains have previously been used in classical bioassay-guided bioprospecting and produce antibiotics or anti-virulence compounds [19,22,27-29,40-45,58]. Strains were routinely grown on Marine Agar (Difco 2216) and in Marine Broth (Difco 2216).

### Genomic DNA isolation and Sequencing

High purity genomic DNA was extracted by successive phenol:chloroform:isoamyl-alcohol purification steps followed by precipitation with isopropanol, treatment with RNase and a final purification and precipitation step [81]. Quantification was done in 1% agarose gel electrophoresis, NanoDrop Spectrometer (Saveen Werner, Sweden) and Qubit 2.0 Analyser (Invitrogen, United Kingdom). Sequencing of the genomes was performed by Beijing Genomic Institute (Shenzhen, China). Libraries of 500 bp were used for 100 bp paired-end sequencing of genomes using the Illumina sequencing technology on a HiSeq2000 with a minimum coverage of 100. Genomic DNA sequences were assembled in contigs using CLC Genomic Workbench (CLC Bio, Aarhus, Denmark). All the genomes had a coverage of 75x or higher. All of them were submitted to the National Center for Biotechnology Information (NCBI) database under the accession numbers AUXW00000000, JMIB00000000, APME00000000, AQCH00000000, CP002972, CP002973, CP002974, CP002975, JXXR00000000, JXXS00000000, JXXT00000000, JXXU00000000, JXXV00000000, JXXW00000000, JXXX00000000, JXXY00000000, JXXZ00000000, JXYA00000000, JXYB00000000, JXYC00000000, JXYD00000000, JXYE00000000, JXYF00000000, JXYG00000000.

### Bioinformatic analysis

The draft genomes were annotated using RAST [49] and submitted to secondary metabolite gene cluster analysis using antiSMASH 2.0 [47], NapDos [38], NP.search [39], as well as to the bacteriocin-specific software BAGEL 3 [37]. Following RAST annotation, a homology search was conducted on the *ferric-iron uptake regulator* gene *fur* and an annotation-based search was performed for genes encoding, chitinases and the chitin catabolic cascade sensor gene *chiS*.

### Verification of antibacterial activity

The strains were re-tested for their antibacterial activity, as previously described [19]. Briefly, strains to be tested were grown in Marine Agar (Difco 2216) for 24 – 48 h and one colony was spotted in plates of artificial seawater agar with 3% Instant Ocean (IO; Aquarium Systems Inc., Sarrebourg, France) containing *Vibrio anguillarum* strain 90-11-287 serotype O1 [82] or *Staphylococcus aureus* strain 8325 [83] embedded. The plates were incubated and observed for clearing zones in the agar.

### Production of acyl homoserine lactones

Production of acyl homoserine lactone (AHL) compounds was analysed using two AHL monitor systems *Agrobacterium tumefaciens* NT1(pZLR4) [84] and *Chromobacterium violaceum* CV026 [85] as described by Ravn et al. [86]. The strains were grown in 10 mL ½YTSS or sea salt medium (1.5% sea salt, 0.3% casamino acids, 0.4% glucose) in 50 mL Falcon tubes for 48 hours at 200 rpm and room temperature and extracted with 10 mL ethyl acetate acidified with 1% formic acid. The extract was dried under nitrogen, resuspended in 0.5 mL ethyl acetate containing 1% formic acid and stored at −20°C. The extracts were tested with the AHL-reporter strains in a plate well assay [87].

### Siderophore activity

Siderophore activity was tested using the liquid CAS assay [69]. The marine strains were grown in 10 mL sea salt medium or ½YTSS in 50 mL Falcon tubes at 25°C and 200 rpm for 24 and 48 hours at room temperature. 1 mL of culture was centrifuged for 5 min at 12,100 × g and the supernatant was mixed with CAS solution in a 1:1 ratio. Colour change from blue to orange indicating siderophore activity was observed after 5 min and 24 h.

### Chitinase activity

Chitinase activity was tested on chitin containing agar plates. Strains were grown on Marine Agar (Difco 2216) for 24 – 48 h and one colony was spotted on plates containing 20 g/L sea salts, 3 g/L casamino acids, 0.08% hydrolyzed chitin, 20 g/L agar. The plates were incubated for 72 h and chitinase activity monitored at 24, 48 and 72 h. The natural turbidity of the media due to chitin allows the visual evaluation of chitin degradation, which leads to clearance of the media. Chitinase activity was graded qualitatively: low chitinase activity (<1.0 mm) zones were scored with one plus, medium chitinase activity zones (1.0 mm – 3.0 mm) with two pluses, and strong chitinase activity (>3.0 mm) with three plusses.

## Abbreviations

NRPS: Non-ribosomal peptide synthase
PKS: Polyketide synthase
KS-domains: Keto-synthase domains
C-domains: Condensation domains
RAST: Rapid Annotation using Subsystem Technology
PFAM: Protein families database
ORF: Open reading frame
profile-HMMs: Profile hidden Markov models
AHL: Acyl homoserine lactone
QS: Quorum sensing
HSL: Homoserine lactone
CAS: Chrome azurol S
Fur: Ferric-iron uptake regulator
ChiS: Chitin catabolic cascade sensor histidine kinase
CBP: Chitin binding protein

## References

[1] Xu J, Hagler A (2002). Chemoinformatics and drug discovery. Molecules.

[2] Scheffler RJ, Colmer S, Tynan H, Demain AL, Gullo VP (2013). Antimicrobials, drug discovery, and genome mining. Appl Microbiol Biotechnol.

[3] Zerikly M, Challis GL (2009). Strategies for the discovery of new natural products by genome mining. Chembiochem.

[4] Newman DJ, Cragg GM (2007). Natural products as sources of new drugs over the last 25 years. J Nat Prod.

[5] Moore BS (2005). Biosynthesis of marine natural products: microorganisms (Part A). Nat Prod Rep.

[6] Xiong Z-Q, Wang J-F, Hao Y-Y, Wang Y (2013). Recent advances in the discovery and development of marine microbial natural products. Mar Drugs.

[7] Meier JL, Burkart MD (2009). The chemical biology of modular biosynthetic enzymes. Chem Soc Rev.

[8] Bérdy J (2012). Thoughts and facts about antibiotics: where we are now and where we are heading. J Antibiot..

[9] Yu D, Xu F, Valiente J, Wang S, Zhan J (2013). An indigoidine biosynthetic gene cluster from *Streptomyces chromofuscus* ATCC 49982 contains an unusual IndB homologue. J Ind Microbiol Biotechnol.

[10] Li B, Walsh CT (2010). Identification of the gene cluster for the dithiolopyrrolone antibiotic holomycin in *Streptomyces clavuligerus*. Proc Natl Acad Sci U S A.

[11] Udwary DW, Zeigler L, Asolkar RN, Singan V, Lapidus A, Fenical W (2007). Genome sequencing reveals complex secondary metabolome in the marine actinomycete *Salinispora tropica*. Proc Natl Acad Sci U S A.

[12] Yin H, Xiang S, Zheng J, Fan K, Yu T, Yang X (2012). Induction of holomycin production and complex metabolic changes by the *argR* mutation in *Streptomyces clavuligerus* NP1. Appl Environ Microbiol.

[13] Li B, Walsh CT (2011). *Streptomyces clavuligerus* HlmI is an intramolecular disulfide-forming dithiol oxidase in holomycin biosynthesis. Biochemistry.

[14] Bhattacharya D, Nagpure A, Gupta RK (2007). Bacterial chitinases: properties and potential. Crit Rev Biotechnol.

[15] Ling LL, Schneider T, Peoples AJ, Spoering AL, Engels I, Conlon BP (2015). A new antibiotic kills pathogens without detectable resistance. Nature.

[16] Ziemert N, Lechner A, Wietz M, Millán-Aguiñaga N, Chavarria KL, Jensen PR (2014). Diversity and evolution of secondary metabolism in the marine actinomycete genus *Salinispora*. Proc Natl Acad Sci U S A.

[17] Zhao X-Q (2011). Genome-based studies of marine microorganisms to maximize the diversity of natural products discovery for medical treatments. Evid Based Complement Alternat Med.

[18] Wietz M, Duncan K, Patin NV, Jensen PR (2013). Antagonistic interactions mediated by marine bacteria: the role of small molecules. J Chem Ecol.

[19] Gram L, Melchiorsen J, Bruhn JB (2010). Antibacterial activity of marine culturable bacteria collected from a global sampling of ocean surface waters and surface swabs of marine organisms. Mar Biotechnol (NY).

[20] Fenical W (1993). Chemical studies of marine bacteria: developing a new resource. Chem Rev..

[21] Lane AL, Moore BS (2011). A sea of biosynthesis: marine natural products meet the molecular age. Nat Prod Rep.

[22] Wietz M, Mansson M, Vynne NG, Gram L. Small molecule antibiotics from marine bacteria and strategies to prevent rediscovery of known compounds. In: Edited by Kim S. Marine microbiology : bioactive compounds and biotechnological applications. Wiley-VCH Verlag GmbH & Co. KGaA; 2013. p. 127-59.

[23] Wilson MC, Mori T, Rückert C, Uria AR, Helf MJ, Takada K (2014). An environmental bacterial taxon with a large and distinct metabolic repertoire. Nature.

[24] Beer LL, Moore BS (2007). Biosynthetic convergence of salinosporamides A and B in the marine actinomycete *Salinispora tropica*. Org Lett.

[25] Feling R, Buchanan G (2003). Salinosporamide A: a highly cytotoxic proteasome inhibitor from a novel microbial source, a marine bacterium of the new genus Salinospora. Angew Chemie..

[26] Still PC, Johnson TA, Theodore CM, Loveridge ST, Crews P (2014). Scrutinizing the scaffolds of marine biosynthetics from different source organisms: Gram-negative cultured bacterial products enter center stage. J Nat Prod.

[27] Månsson M, Phipps RK, Gram L, Munro MHG, Larsen TO, Nielsen KF (2010). Explorative Solid-Phase Extraction (E-SPE) for accelerated microbial natural product discovery, dereplication, and purification. J Nat Prod.

[28] Mansson M, Gram L, Larsen TO (2011). Production of bioactive secondary metabolites by marine vibrionaceae. Mar Drugs.

[29] Wietz M, Mansson M, Gotfredsen CH, Larsen TO, Gram L (2010). Antibacterial compounds from marine Vibrionaceae isolated on a global expedition. Mar Drugs.

[30] Weber T (2014). *In silico* tools for the analysis of antibiotic biosynthetic pathways. Int J Med Microbiol.

[31] Fedorova ND, Moktali V, Medema MH, Keller NP, Turner G (2012). Bioinformatics approaches and software for detection of secondary metabolic gene clusters. Fungal secondary metabolism: methods and protocols.

[32] Seyedsayamdost MR (2014). High-throughput platform for the discovery of elicitors of silent bacterial gene clusters. Proc Natl Acad Sci U S A.

[33] Weber T, Rausch C, Lopez P, Hoof I, Gaykova V, Huson DH (2009). CLUSEAN: a computer-based framework for the automated analysis of bacterial secondary metabolite biosynthetic gene clusters. J Biotechnol.

[34] Rausch C, Weber T, Kohlbacher O, Wohlleben W, Huson DH (2005). Specificity prediction of adenylation domains in Nonribosomal Peptide Synthetases (NRPS) using Transductive Support Vector Machines (TSVMs). Nucleic Acids Res.

[35] Röttig M, Medema MH, Blin K, Weber T, Rausch C, Kohlbacher O (2011). NRPSpredictor2--a web server for predicting NRPS adenylation domain specificity. Nucleic Acids Res.

[36] Minowa Y, Araki M, Kanehisa M (2007). Comprehensive analysis of distinctive polyketide and nonribosomal peptide structural motifs encoded in microbial genomes. J Mol Biol.

[37] Van Heel AJ, de Jong A, Montalbán-López M, Kok J, Kuipers OP (2013). BAGEL3: automated identification of genes encoding bacteriocins and (non-)bactericidal posttranslationally modified peptides. Nucleic Acids Res.

[38] Ziemert N, Podell S, Penn K, Badger JH, Allen E, Jensen PR (2012). The natural product domain seeker NaPDoS: a phylogeny based bioinformatic tool to classify secondary metabolite gene diversity. PLoS One.

[39] Li MHT, Ung PMU, Zajkowski J, Garneau-Tsodikova S, Sherman DH (2009). Automated genome mining for natural products. BMC Bioinformatics.

[40] Mansson M, Nielsen A, Kjærulff L, Gotfredsen CH, Wietz M, Ingmer H (2011). Inhibition of virulence gene expression in *Staphylococcus aureus* by novel depsipeptides from a marine *Photobacterium*. Mar Drugs.

[41] Nielsen A, Månsson M, Bojer MS, Gram L, Larsen TO, Novick RP (2014). Solonamide B inhibits quorum sensing and reduces *Staphylococcus aureus* mediated killing of human neutrophils. PLoS One.

[42] Kjaerulff L, Nielsen A, Mansson M, Gram L, Larsen TO, Ingmer H (2013). Identification of four new *agr* quorum sensing-interfering cyclodepsipeptides from a marine *Photobacterium*. Mar Drugs.

[43] Nielsen A, Mansson M, Wietz M, Varming AN, Phipps RK, Larsen TO (2012). Nigribactin, a novel siderophore from *Vibrio nigripulchritudo*, modulates *Staphylococcus aureus* virulence gene expression. Mar Drugs.

[44] Vynne NG, Månsson M, Nielsen KF, Gram L (2011). Bioactivity, chemical profiling, and 16S rRNA-based phylogeny of *Pseudoalteromonas* strains collected on a global research cruise. Mar Biotechnol (NY).

[45] Vynne NG, Mansson M, Gram L (2012). Gene sequence based clustering assists in dereplication of *Pseudoalteromonas luteoviolacea* strains with identical inhibitory activity and antibiotic production. Mar Drugs.

[46] Medema MH, Blin K, Cimermancic P, de Jager V, Zakrzewski P, Fischbach MA (2011). antiSMASH: rapid identification, annotation and analysis of secondary metabolite biosynthesis gene clusters in bacterial and fungal genome sequences. Nucleic Acids Res.

[47] Blin K, Medema MH, Kazempour D, Fischbach MA, Breitling R, Takano E (2013). antiSMASH 2.0--a versatile platform for genome mining of secondary metabolite producers. Nucleic Acids Res.

[48] Overbeek R, Olson R, Pusch GD, Olsen GJ, Davis JJ, Disz T (2014). The SEED and the rapid annotation of microbial genomes using Subsystems Technology (RAST). Nucleic Acids Res.

[49] Aziz RK, Bartels D, Best AA, DeJongh M, Disz T, Edwards RA (2008). The RAST Server: rapid annotations using subsystems technology. BMC Genomics.

[50] Omura S, Ikeda H, Ishikawa J, Hanamoto A, Takahashi C, Shinose M (2001). Genome sequence of an industrial microorganism *Streptomyces avermitilis*: deducing the ability of producing secondary metabolites. Proc Natl Acad Sci U S A.

[51] Bentley SD, Chater KF, Cerdeño-Tárraga A-M, Challis GL, Thomson NR, James KD (2002). Complete genome sequence of the model actinomycete *Streptomyces coelicolor* A3(2). Nature.

[52] Buchan A, González J, Moran M (2005). Overview of the marine roseobacter lineage. Appl Environ.

[53] Wagner-Döbler I, Biebl H (2006). Environmental biology of the marine Roseobacter lineage. Annu Rev Microbiol.

[54] Porsby CH, Nielsen KF, Gram L (2008). *Phaeobacter* and *Ruegeria* species of the Roseobacter clade colonize separate niches in a Danish Turbot (*Scophthalmus maximus*)-rearing farm and antagonize Vibrio anguillarum under different growth conditions. Appl Environ Microbiol.

[55] Seyedsayamdost MR, Carr G, Kolter R, Clardy J (2011). Roseobacticides: small molecule modulators of an algal-bacterial symbiosis. J Am Chem Soc.

[56] Brinkhoff T, Bach G, Heidorn T, Liang L, Schlingloff A, Simon M (2004). Antibiotic Production by a Roseobacter Clade-Affiliated Species from the German Wadden Sea and Its Antagonistic Effects on Indigenous Isolates. Appl Environ Microbiol.

[57] Geng H, Bruhn JB, Nielsen KF, Gram L, Belas R (2008). Genetic dissection of tropodithietic acid biosynthesis by marine roseobacters. Appl Environ Microbiol.

[58] Wietz M, Månsson M, Gram L (2011). Chitin stimulates production of the antibiotic andrimid in a *Vibrio coralliilyticus* strain. Environ Microbiol Rep.

[59] Jin M, Fischbach MA, Clardy J (2006). A biosynthetic gene cluster for the acetyl-CoA carboxylase inhibitor andrimid. J Am Chem Soc.

[60] Gauthier MJ (1976). Morphological, physiological, and biochemical characteristics of some violet-pigmented bacteria isolated from seawater. Can J Microbiol.

[61] Laatsch H, Pudleiner H (1989). Marine bakterien, I: synthese von pentabrompseudilin, einem phenylpyrrol aus Alteromonas luteoviolaceus. Liebigs Ann Chem..

[62] Moore BS (2006). Biosynthesis of marine natural products: macroorganisms (Part B). Nat Prod Rep.

[63] Conway KR, Boddy CN (2013). ClusterMine360: a database of microbial PKS/NRPS biosynthesis. Nucleic Acids Res.

[64] Bruhn JB, Nielsen KF, Hjelm M, Hansen M, Bresciani J, Schulz S (2005). Ecology, inhibitory activity, and morphogenesis of a marine antagonistic bacterium belonging to the Roseobacter clade. Appl Environ Microbiol.

[65] Holden MTG, Chhabra SR, De Nys R, Stead P, Bainton NJ, Hill PJ (1999). Quorum-sensing cross talk: isolation and chemical characterization of cyclic dipeptides from *Pseudomonas aeruginosa* and other gram-negative bacteria. Mol Microbiol.

[66] Rasmussen BB, Nielsen KF, Machado H, Melchiorsen J, Gram L, Sonnenschein EC (2014). Global and phylogenetic distribution of quorum sensing signals, acyl homoserine lactones, in the family of vibrionaceae. Mar Drugs.

[67] Vynne NG (2011). Bioactivity and phylogeny of the marine bacterial genus *Pseudoalteromonas*.

[68] Sandy M, Butler A (2009). Microbial iron acquisition: marine and terrestrial siderophores. Chem Rev.

[69] Schwyn B, Neilands JB (1987). Universal chemical assay for the detection and determination of siderophores. Anal Biochem.

[70] Hider RC, Kong X (2010). Chemistry and biology of siderophores. Nat Prod Rep.

[71] Rudolph G, Hennecke H, Fischer H-M (2006). Beyond the Fur paradigm: iron-controlled gene expression in rhizobia. FEMS Microbiol Rev.

[72] Williams KP, Gillespie JJ, Sobral BWS, Nordberg EK, Snyder EE, Shallom JM (2010). Phylogeny of gammaproteobacteria. J Bacteriol.

[73] Sawabe T, Ogura Y, Matsumura Y, Feng G, Amin AR, Mino S (2013). Updating the *Vibrio* clades defined by multilocus sequence phylogeny: proposal of eight new clades, and the description of *Vibrio tritonius* sp. nov. Front Microbiol.

[74] Pascual J, Macián MC, Arahal DR, Garay E, Pujalte MJ (2010). Multilocus sequence analysis of the central clade of the genus Vibrio by using the *16S rRNA*, *recA*, *pyrH*, *rpoD*, *gyrB*, *rctB* and *toxR* genes. Int J Syst Evol Microbiol.

[75] Thompson FL, Gevers D, Thompson CC, Dawyndt P, Naser S, Hoste B (2005). Phylogeny and molecular identification of vibrios on the basis of multilocus sequence analysis. Appl Environ Microbiol..

[76] Meibom KL, Li XB, Nielsen AT, Wu C-Y, Roseman S, Schoolnik GK (2004). The *Vibrio cholerae* chitin utilization program. Proc Natl Acad Sci U S A.

[77] Li X, Roseman S (2004). The chitinolytic cascade in Vibrios is regulated by chitin oligosaccharides and a two-component chitin catabolic sensor/kinase. Proc Natl Acad Sci U S A.

[78] Frederiksen RF, Paspaliari DK, Larsen T, Storgaard BG, Larsen MH, Ingmer H (2013). Bacterial chitinases and chitin-binding proteins as virulence factors. Microbiology.

[79] Souza CP, Almeida BC, Colwell RR, Rivera ING (2011). The importance of chitin in the marine environment. Mar Biotechnol (NY).

[80] Stauder M, Huq A, Pezzati E, Grim CJ, Ramoino P, Pane L (2012). Role of GbpA protein, an important virulence-related colonization factor, for *Vibrio cholerae*’s survival in the aquatic environment. Environ Microbiol Rep.

[81] Sambrook J, Russel DW (2001). Molecular cloning: a laboratory manual.

[82] Skov MN, Pedersen K, Larsen JL (1995). Comparison of pulsed-field gel electrophoresis, ribotyping, and plasmid profiling for typing of *Vibrio anguillarum* serovar O1. Appl Environ Microbiol.

[83] Novick R, Morse S (1967). In vivo transmission of drug resistance factors between strains of Staphylococcus aureus. J Exp Med.

[84] Cha C, Gao P, Chen YC, Shaw PD, Farrand SK (1998). Production of acyl-homoserine lactone quorum-sensing signals by gram-negative plant-associated bacteria. Mol Plant Microbe Interact.

[85] Mcclean KH, Winson MK, Fish L, Taylor A, Chhabra SR, Camara M (1997). Quorum sensing and *Chromobacterium violaceum*: exploitation of violacein production and inhibition for the detection of N -acylhomoserine lactones.

[86] Ravn L, Christensen AB, Molin S, Givskov M, Gram L (2001). Methods for detecting acylated homoserine lactones produced by Gram-negative bacteria and their application in studies of AHL-production kinetics. J Microbiol Methods.

[87] Gram L, Grossart H (2002). Possible quorum sensing in marine snow bacteria: production of acylated homoserine lactones by Roseobacter strains isolated from marine snow. Appl.

